# Ultrasonic Sensors and Ultrasonic Signal Processing

**DOI:** 10.3390/s26216941

**Published:** 2026-11-03

**Authors:** Ming Zhu

**Affiliations:** School of Electronic Information and Communications, Huazhong University of Science and Technology, Wuhan 430074, China; zhuming@hust.edu.cn

## 1. Introduction

Ultrasonic sensing supports nondestructive testing, material characterization, motion and flow measurement, and biomedical imaging. Its performance depends on both sensor design and signal interpretation. Detecting weak responses, resolving overlapping signals, and balancing different measurement requirements remain challenges across these applications.

The Special Issue “Ultrasonic Sensors and Ultrasonic Signal Processing” brings together eight research articles on sensing structures, array design, signal processing, and imaging. This Editorial introduces the contributions and discusses their connections through three themes: improving measurement conditions, extracting useful information from complex signals, and addressing the limitations of a single measurement configuration.

## 2. Overview of Contributions

Table 1 summarizes the research areas, focuses, methods, and applications of the eight contributions.

In Contribution 1, Shevelko et al. developed an ultrasonic gyroscopic sensing element using a layered standing-wave structure to enhance the rotation-dependent response. Theoretical and numerical results were compared with prototype measurements. Rotation experiments identified an output amplitude related to angular velocity, demonstrating the feasibility of the sensing principle.

In Contribution 2, Zeng et al. optimized cylindrical ultrasonic arrays for casing inspection. A neural-network model and multi-objective optimization were used to balance beam width, side-lobe responses, and sound-pressure uniformity. Acoustic field simulations evaluated the selected designs; device fabrication and experimental validation remain future work.

In Contribution 3, Zhou et al. investigated ultrasonic inspection of laser welds in titanium alloy plates. Simulations examined frequency-dependent defect and background responses. A calibrated system with a 5 MHz shear-wave angle transducer detected four prepared internal defects, including an equivalent size of 1 mm, with a mean absolute localization error of 0.438 mm.

In Contribution 4, Leng et al. developed a learning framework for damage localization in molten salt receiver tubes. Following initial detection, the model estimated defect-related echo boundaries from time–frequency images. Joint optimization of signal representation and network parameters improved interval localization in experimental data. The study also distinguished signal-domain prediction errors from errors in conversion to physical distance.

In Contribution 5, Dai et al. estimated grain size distributions in GH4099 nickel-based superalloy using ultrasonic signals and deep learning. The method retained amplitude and phase information and averaged neighboring predictions to match the metallographic sampling region. Experiments showed that changing probe frequency reduced prediction accuracy and that transfer training improved adaptation.

In Contribution 6, Niu et al. combined a polyvinylidene fluoride film transmitter, simultaneous bidirectional reception, and signal processing for ultrasonic wind measurement. Signal decomposition, filtering, and time-delay estimation recovered transit times from distorted waveforms. Wind tunnel tests over 0–60 m/s showed improved robustness relative to the compared methods, although substantial errors remained at high speeds.

In Contribution 7, Liu and Emelianov used separate transducers for excitation and motion tracking in shear wave elasticity imaging. This arrangement allowed the two tasks to use different frequencies. Phantom experiments showed that higher-frequency tracking improved signal-to-noise ratio, contrast-to-noise ratio, spatial resolution, and measurement precision in the tested configurations.

In Contribution 8, Huber et al. combined magnetomotive ultrasound and passive cavitation mapping to image superparamagnetic iron oxide nanoparticles. Phantom experiments demonstrated complementary sensitivity: magnetomotive ultrasound detected displacement associated with tissue-bound particles, while passive cavitation mapping detected acoustic emissions from flowing suspensions under focused ultrasound exposure.

## 3. Conclusions

The eight articles in this Special Issue explore ultrasonic sensing and signal processing through approaches spanning device design, measurement, and imaging. Across different applications, the contributions demonstrate ways to advance ultrasonic measurement by improving signal acquisition, extracting useful information, and combining complementary observations.

The contributions are connected by the dependence of measurement performance on how signals are acquired, interpreted, and used across different measurement tasks. Studies of sensing structures, array parameters, and inspection frequency seek responses better suited to the measurement objective, while work on damage localization, grain characterization, and wind measurement extracts target information from complex signals. Separating excitation from tracking and combining different imaging methods further illustrate how task allocation and complementary information can address the limitations of a single configuration. These connections provide a basis for exchanging methodological insights across applications.

Building on these contributions, future research can jointly develop devices and signal processing methods for specific measurement objectives, extend their applicability across materials, probes, and operating conditions, and explore complementary measurements in practical systems. The design approaches and research methods presented in this Special Issue offer useful starting points for further advances in ultrasonic sensing, nondestructive testing, and biomedical imaging.

## Figures and Tables

**Table 1:** Overview of the contributions to the Special Issue.

Contribution No.	Research Area	Focus	Type of Research	Application Area
1	Ultrasonic sensor design	Standing-wave sensing element; angular velocity response.	Theoretical modeling, numerical simulation, and prototype experiments.	Inertial sensing
2	Ultrasonic array design	Cylindrical array parameters; multi-objective acoustic field optimization.	Computational optimization and acoustic field simulation.	Casing inspection
3	Ultrasonic nondestructive testing	Frequency selection; internal weld defect detection and localization.	Numerical simulation and experimental testing.	Titanium alloy weld inspection
4	Ultrasonic signal processing	Guided-wave signal representation; defect interval localization.	Algorithm development and experimental data evaluation.	Molten salt receiver tube inspection
5	Ultrasonic material characterization	Grain size distribution prediction; adaptation to probe frequency changes.	Method development and specimen experiments.	Nickel-based superalloy characterization
6	Ultrasonic flow measurement	Acoustic transit-time estimation; noise suppression and waveform recovery.	System development and wind tunnel experiments.	Wind speed measurement
7	Biomedical ultrasound imaging	Separate excitation and tracking; elasticity imaging performance.	Experimental research using tissue-mimicking phantoms.	Tissue elasticity imaging
8	Biomedical ultrasound imaging	Complementary magnetomotive and passive cavitation imaging.	Experimental research using tissue and flow phantoms.	Nanoparticle distribution imaging

